# A Study of Using Natural Sorbent to Reduce Iron Cations from Aqueous Solutions

**DOI:** 10.3390/ijerph17103686

**Published:** 2020-05-23

**Authors:** Iveta Pandová, Miroslav Rimár, Anton Panda, Jan Valíček, Milena Kušnerová, Marta Harničárová

**Affiliations:** 1 Faculty of Manufacturing Technologies, Technical University of Košice, 080 01 Prešov, Slovakia; iveta.pandova@tuke.sk (I.P.); miroslav.rimar@tuke.sk (M.R.); anton.panda@tuke.sk (A.P.); 2 Faculty of Engineering, Slovak University of Agriculture, 949 76 Nitra, Slovakia; jan.valicek@uniag.sk or; 3 Faculty of Technology, College of Technology and Business in České Budějovice, 370 01 České Budějovice, Czech Republic; kusnerova.milena@mail.vstecb.cz

**Keywords:** zeolite clinoptilolite, ferric cations, wastewater, sorption, adsorption isotherm

## Abstract

Iron is an essential trace element, but at high doses, this element may pose a health risk. Wastewater from iron ore mining, steel production, and metal processing, among other heavy metals, also contains high concentrations of iron (Fe^3+^). The use of sorption on natural materials is a potential alternative to conventional methods for removing iron ions, also because of low cost. The methods presented in this article are based on the study of kinetic properties and the acquisition of adsorption isotherms, which are one of the most important characteristics of adsorption mechanisms. The course of sorption is analyzed according to the Freundlich sorption isotherm model. Isotherm parameters are evaluated using experimental results of ferric cation sorption. The results presented relate to the investigation of natural zeolite-clinoptilolite as a ferric cation sorbent, providing a measurement of the sorption kinetics as well as the observed sorption parameters of iron cations from aqueous media. The optimal time for equilibrium in the adsorption system is determined from the kinetic dependencies. The dependence of the achieved equilibrium concentration on the initial concentration of the solution was also expressed, both graphically and analytically. The new prediction model was compared with the traditional Freundlich model. Finally, adsorption isotherms tested under laboratory conditions for a practical application can be recommended for the preliminary examination of the possible technological use of natural zeolite in the wastewater treatment process.

## 1. Introduction

Contamination of soils, groundwater, surface waters, and air with hazardous toxic chemicals presents significant problems for human health and the environment. In particular, heavy metals are considered hazardous pollutants [1].

Their presence in wastewater comes from various industrial processes due to the development of industrial technologies. Various adsorbents, such as activated carbon [2], astragalus [3], carbon nanotubes [4,5], and a large number of bio-sorbents [6] have been used in various studies to remove pollutants, especially heavy metals from wastewater.

However, new and efficient adsorbents with local availability in addition to economic suitability are still needed. Heavy metals are not biodegradable and tend to accumulate in living organisms. With regard to the impacts on human health, each contaminant causes different effects and symptoms. Wastewater from iron ore mining, steel production, and metal processing contains, in addition to heavy metals, high concentrations of iron (Fe^3+^) in the range of 50–95,000 mg × dm^−3^. According to the World Health Organization, iron is one of the metallic elements that causes significant environmental damage. Iron, along with some other heavy metals, is an essential trace element, which means that it is crucial for the human body and other living organisms. However, at high doses, iron can pose a health risk and can even become a poison. The first symptom is abdominal pain. If the iron content in drinking water exceeds 0.2 mg × dm^−3^, it may cause digestive problems. Iron in water is very toxic to fish. In addition, the high content of ferric cations causes turbidity, unpleasant taste, and odor. It also causes difficulties in distribution systems by promoting bacterial growth, resulting in pipe clogging and increasing flow characteristics. For this reason, the presence of iron ions in both waste and drinking water is regulated.

## 2. Trends in the Use of Sorbents for the Removal of Iron Ions from Aqueous Solutions

The principle of the methods used to remove iron from water is to oxidize the initially dissolved iron to undissolved compounds, which can be removed by one or two-step separation. The oxidation and hydrolysis of these compounds take place under strictly defined conditions. Precipitation processes can achieve the removal of Fe^3+^ ions, but their residual concentrations are still detectable in the liquid phase. Therefore, there is a need for purification, for example, by ion exchange or sorption. Techniques commonly used to reduce iron levels are also associated with other problems, such as the formation of toxic intermediates of chemical substances resulting from chemical reactions, the high operating costs necessary to maintain physical and chemical conditions, and the relatively long time of the biological treatment technique. The development of sorption materials and technologies for the effective removal of salts and unwanted contaminants from water is a promising way to address the global water crisis [7].

Zeolites with excellent adsorption and ion exchange capacity have found wide application in these areas. The most commonly used sorbent is activated carbon and synthetic zeolites. These sorbents are relatively expensive. In this respect, natural zeolites, whose abundant resources are located in various regions of the Earth, might be more promising. Zeolites are considered worldwide to be widely applicable in various fields of science and technology, as well as environmental science. According to literature, Y-type zeolite is suitable for the removal of heavy metal cations from industrial water [8,9]. The study of the removal of iron ions by sorption is addressed by several authors who analyze the equilibrium adsorption data in their work using the Langmuir and Freundlich isotherm model [10,11,12,13].

In order to improve the ability of the zeolites to absorb heavy metals, iron oxides are used. They are either added to the reaction mixture during the zeolite synthesis itself or deposited on the surface of the zeolite. Based on various studies, it has been shown that zeolite with iron oxide on the surface has a higher sorption capacity for Cu, Pb, Zn, Mn, and Cd. The reason for this phenomenon is an increase in the Fe-OH binding sites contained in iron oxides. Some heavy metals can adsorb to zeolite not only by ion exchange, but also by binding to specific sites containing AlOH, SiOH, or FeOH [14].

Several models can be used to describe the adsorption of pollutants from aqueous solutions. One is the pseudo-second-order model. The advantage of using this model is that it is not necessary to know the equilibrium capacity from the experiments, as it can be calculated from the model [15]. A very frequently used method of removing iron from water is by contact filtration, namely filtration on manganese filters. The addition of potassium permanganate forms a coating on the surface of the filter cartridge, which serves as an oxidation catalyst. The oxidation state of the MnO_x_ filler coating plays an important role in the removal of dissolved iron [16].

Techniques commonly used to reduce iron levels are associated with problems such as, in particular, the formation of toxic chemical intermediates as a result of chemical reactions, high operating costs required to maintain physicochemical conditions, as well as relatively long time to implement biological treatment techniques. Therefore, techniques applying the action of clinoptilolite, which has properties in comparison with conventional materials used in treatment techniques, predetermine this natural material for use in water treatment processes, are preferred. Clinoptilolite, in combination with silica sand, is more effective in removing iron, aluminum, copper, ammoniacal nitrogen even in slow sand filtration. It allowed increasing the filtration rate up to four times. Clinoptilolite has a lower specific weight compared to silica sand. Its porosity and sludge capacity is 1.5 times higher than the values of filter sand, meeting the requirements for granular filter materials [16]. The publication [13] states that nanomaterials with a higher adsorption surface and surface treatment by incorporating specific functional groups (materials based on metal oxides, dendrimers, zeolites) are more effective in water protection. Of the wide range of ion exchangers and sorbents, zeolites play an important role due to their high affinity for heavy metals as well as the ion exchange capacity. Zeolites suitable in the treatment of water to remove heavy metal ions include both synthetic NaY and Y zeolites. Clinoptilolite is the most commonly used of natural zeolites. Zeolites have a three-dimensional spatial structure made up of [SiO_4_]^4−^ and [AlO_4_]^5−^ [17] tetrahedrons. The backbone of the zeolites consists of the [SiO_4_]^4−^ tetrahedron network in which aluminum atoms isomorphically replace part of the silicon atoms. This creates a negative charge, which is compensated by mobile cations of alkali metals or alkaline earth metals [17,18]. The negative charge that arises from the replacement of silicon atoms by aluminum atoms is compensated by the presence of sodium, potassium, and calcium cations. Inside the crystalline system of zeolites, channels of precisely defined dimensions are formed, in which these cations are located. An important feature of zeolites is the ability to exchange these cations for those found in the surrounding environment, including in the aquatic environment. Heavy metal cations removed from aqueous solutions are immobilized on the zeolite by two mechanisms, namely ion exchange and chemisorption. In the treatment of water, the zeolites in their natural, i.e., unmodified form, fulfil the function of cation exchangers on the basis of the property mentioned above [18].

The clinoptilolite-type zeolite as a natural and non-toxic material is ecologically suitable and affordable, and it can be assumed that it will be of interest in the future [19]. Due to its ion-exchange and sorption properties as well as many other properties, it is a suitable material for use in various fields. The structure of clinoptilolite is isomorphic to that of heulandite. Based on a perfectly formed microporous system of channels and cavities, zeolites are considered excellent molecular sieves. These properties are related to their subsequent use in water treatment and purification [8,20]. The most commonly used natural zeolites include clinoptilolite, mordenite, erionite, ferrierite. Clinoptilolite is defined as a mesoporous material that is mostly aggregated by colloidal dispersed nanoparticles up to 30%. Its surface is hydrophilic and has various active centers that can be used in van der Waals and electrostatic interactions with adsorbate [21]. Because of these properties, clinoptilolite is suitable for sorption, ion exchange, and catalytic purposes. Previous studies of this natural material have confirmed its ability to remove copper cations from the aquatic environment as well as some toxic substances from the exhaust gases [22,23]. Clinoptilolite-type zeolites retain their structure over a wide pH range of 1.0–11.5 [24].

Sorption is one of the latest methods of water treatment technology. It is realized through physicochemical mechanisms such as chemisorption, ion exchange, precipitation, and physical adsorption. Physical adsorption is based exclusively on surface physical (electrostatic) forces (e.g., van der Waals forces). The adsorbate (bound substance) molecules are not bound to specific sites on the adsorbent surface. Multiple layers of adsorbate can be formed (multilayer or multimolecular adsorption). During physical adsorption, the molecules of the adsorbed substance do not break down; in particular, the multilayer character of the adsorbed substance is typical. On zeolites, metal cations are immobilized by two mechanisms—ion exchange and chemisorption [18]. In this way, as in other microporous materials, also in zeolites, the ion-exchange sorption between the liquid and solid phase components takes place according to the surface diffusion and the internal diffusion mechanism. The mechanism of sorption of iron cations on a silica sorbent is described in the publication [25].

For the discontinuous arrangement of the process, it is necessary to know the kinetic course of adsorption and adsorption isotherm. Therefore, the main objective of the presented experiments is to obtain these important characteristics of adsorption mechanisms and to explore the possibility of technological use of natural zeolite-clinoptilolite in the process of water purification.

Sorption on synthetic zeolites is known, but the possibility of replacing them with natural ones in water purification is being investigated. The paper investigates the sorption of iron cations in a specific type of natural zeolite, namely clinoptilolite from Nižný Hrabovec, which has a crystalline structure identical to clinoptilolite, i.e., heulandite rich in Si (according to Tschernich).

Based on the performed research, we defined the goal to obtain a new prediction model for the dependence of the achieved equilibrium concentration on the initial concentration of the solution. Based on the fulfilment of this goal, it is possible to predict parameters for the subject group of material that could be used in the application sphere (e.g., for water purifiers).

## 3. Materials and Methods

### 3.1. Natural Zeolite-Clinoptilolite

**Figure 1:**
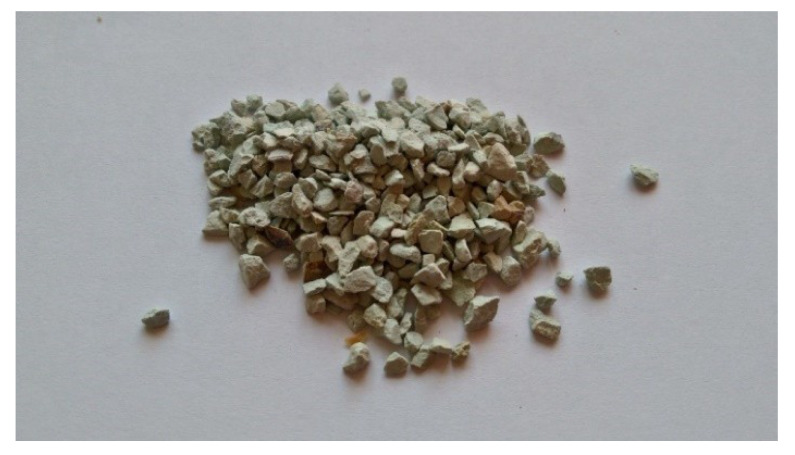
Zeolite-clinoptilolite, fraction 2.5 to 5 mm [23].

In order to investigate the sorption of ferric cations on natural zeolite, laboratory experiments were carried out using natural zeolite-clinoptilolite in granular form with a grain size of 2.5 to 5 mm (Figure 1). Zeolite from this locality contains 84% clinoptilolite, 3%–4% feldspars, 8% cristobalite, 2%–5% quartz, and 13%–30% volcanic glass. The primary cation in natural clinoptilolite from this deposit is potassium. The Si/Al ratio of clinoptilolite from this deposit ranges from 4.0 to 5.2 [26].

The mechanism of the purification of zeolites is the fact that the Si:Al ratio in zeolites determines the size of their pores so that such highly porous materials can selectively remove ions even smaller than 2 nm. The equations of the mechanism can be applied only to the part of the process that has the character of ion exchange. Because sorption in zeolites is caused by the properties of the structure with large spaces (cavities, pores), the sorption process is most often described by isotherms or is modeled. It is difficult to compare results from literature, it often represents contradictory conclusions also because the sorption capacity of a particular zeolite is not entirely determined by the total cation exchange capacity since the specific crystal structure and distribution and availability of cation exchanges have a crucial role in determining the extent of exchange cations in the zeolite [27].

Sorption was performed from model samples. The compound Fe_2_(SO_4_)_3_∙(NH_4_)_2_SO_4_∙24 × H_2_O was used to prepare the model samples. The decrease in Fe^3+^ concentration in the solution in equidistant time intervals was investigated. Absorption photometry using a digital colorimeter (Model AC 114, Optima, Tokyo, Japan) was applied to determine the concentration of ferric cations in aqueous solution quantitatively. Measurements were taken at a wavelength of 660 nm. The mass concentration of ferric cations was determined by the calibration curve method. All experiments were performed three times under the same conditions. All data represent the average of three independent measurements. Sorption isotherms were measured at 22 °C.

### 3.2. Monitoring Kinetic Properties

Monitoring kinetic properties and obtaining adsorption isotherms is one of the most important characteristics of adsorption mechanisms. The course of sorption can be analyzed using several models. The most commonly used is the Langmuir and Freundlich model of the isotherm. Which model more suits the given sorption is determined experimentaly. Verification is carried out graphically or mathematically using the least-squares method.

The dependence of the adsorption capacity *q* on the concentration of the solution at equilibrium can be described by Equation (1) [18]:

(1) $$q=q_{\max}\times \frac{K\times c_{e}}{1+K\times c_{e}}$$

where *q_max_* and *K* are the Langmuir constants that are related to the maximum adsorption capacity and adsorption energy and can be determined experimentally. The experimental values plotted in the coordinates *X_i_* = *c_e,i_*, *Y_i_* = *c_e,i_*_×_ *q_i_*^−1^ then lie on the line. If the Freundlich isotherm *q = K_F ×_ c_e_* ^1/*n*^ is suitable for the expression of the dependence *q* = *f*(*c_e_*), it is possible to translate the line with experimental values plotted in the coordinates *X* = log *c_e_*, *Y* = log *q*. For further calculations, we take the one from both dependencies, for which the plotted points suit the linear relation better. For the qualitative expression of adsorption, it is necessary to know the amount of substance or weight adsorbed by the adsorbent weight unit. The specific adsorption of a particular component can be expressed according to Equation (2) [28]:

(2) $$q=\frac{c_{0}-c_{e}}{m}\times V$$

where *q* is the equilibrium adsorption capacity of adsorbate, *V* is the volume of the solution in contact with the adsorbent, *m* is the mass of adsorbent, *c*_0_ is the initial material or mass concentration of adsorbate in solution, *c_e_* is the concentration of adsorbate during equilibrium. The sorbent efficiency is calculated according to Equation (3) [13,18]:

(3) $$E=\frac{c_{0}-c_{e}}{c_{0}}\times 100$$

where *E* is the sorbent activity, and *c*_0_ is the initial concentration in the solution

The equilibrium concentration value in aqueous solutions depends on the properties of adsorbent used. In order to define the time needed to stabilize the equilibrium in the system, the dependence of adsorbed amount on the time of contact of adsorbent with adsorbate was observed. Laboratory experiments were performed in glass beakers weighing 40 g of adsorbent sample with analytical accuracy, adding 0.25 dm^−3^ of an aqueous solution of ferric cations with an initial concentration of 0.46 g × dm^−3^, 0.56 g × dm^−3^, 0.82 g × dm^−3^, 1.12 g × dm^−3^, 1.28 g × dm^−3^, and 1.40 g × dm^−3^. The model samples were mixed, and the minimum amount necessary for the analytical determination of the ferric cation concentration was taken at precise time intervals. At the same time, an adequate amount of adsorbent was taken. After the separation of the solids, the concentration of cations in the solution was determined by a calibration curve method using photometry.

## 4. Results

### 4.1. Defining Equilibrium in the System

**Figure 2:**
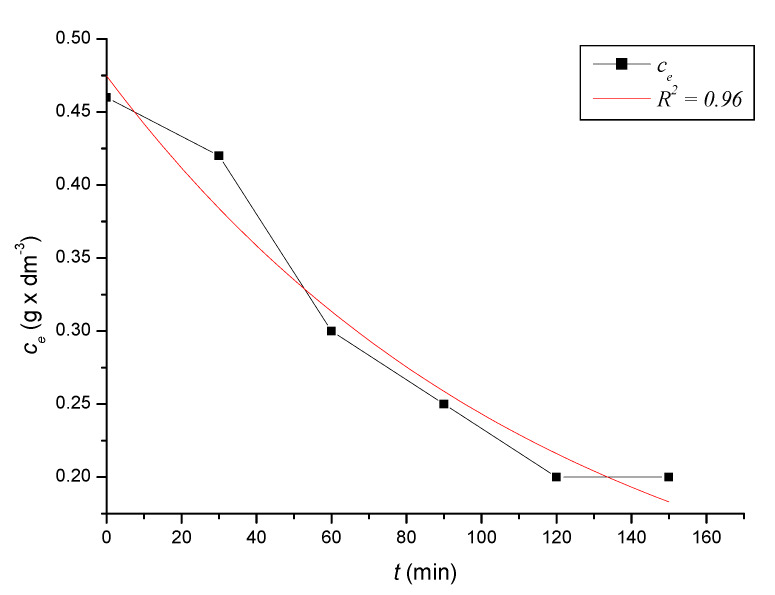
The kinetic course of reducing the concentration of Fe^3+^ cations in a solution with an initial concentration of 0.46 g × dm^−3^ by the action of clinoptilolite.

**Figure 3:**
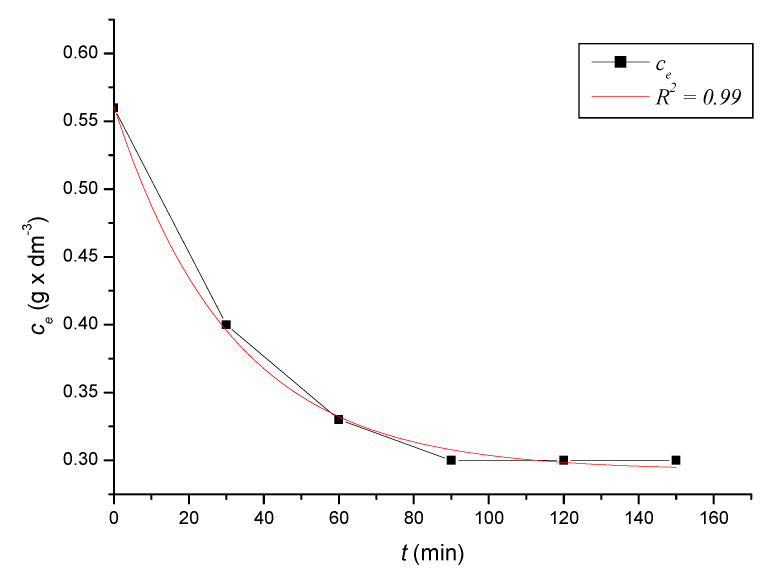
The kinetic course of reducing the concentration of Fe^3+^ cations in a solution with an initial concentration of 0.56 g × dm^−3^ by the action of clinoptilolite.

**Figure 4:**
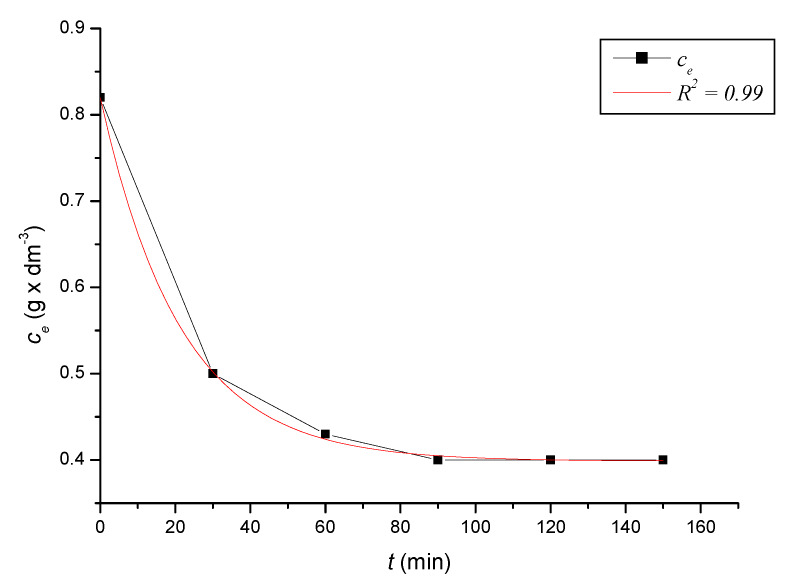
The kinetic course of reducing the concentration of Fe^3+^ cations in a solution with an initial concentration of 0.82 g × dm^−3^ by the action of clinoptilolite.

**Figure 5:**
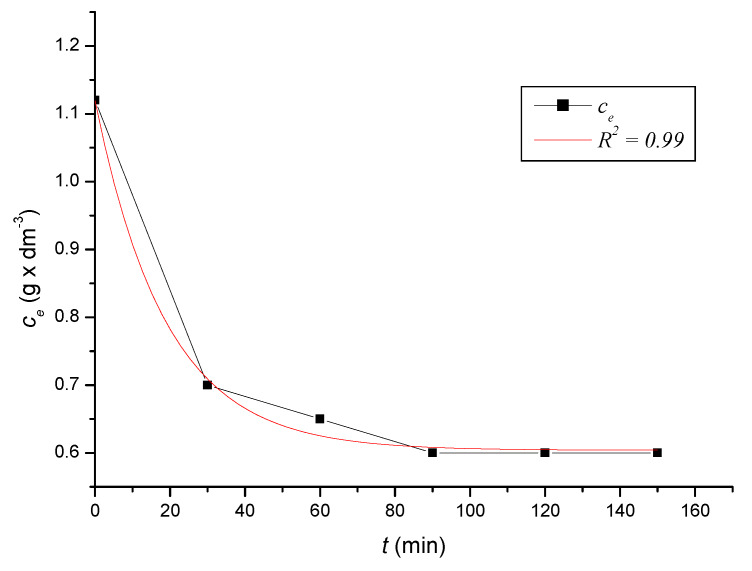
The kinetic course of reducing the concentration of Fe^3+^ cations in a solution with an initial concentration of 1.12 g × dm^−3^ by the action of clinoptilolite.

**Figure 6:**
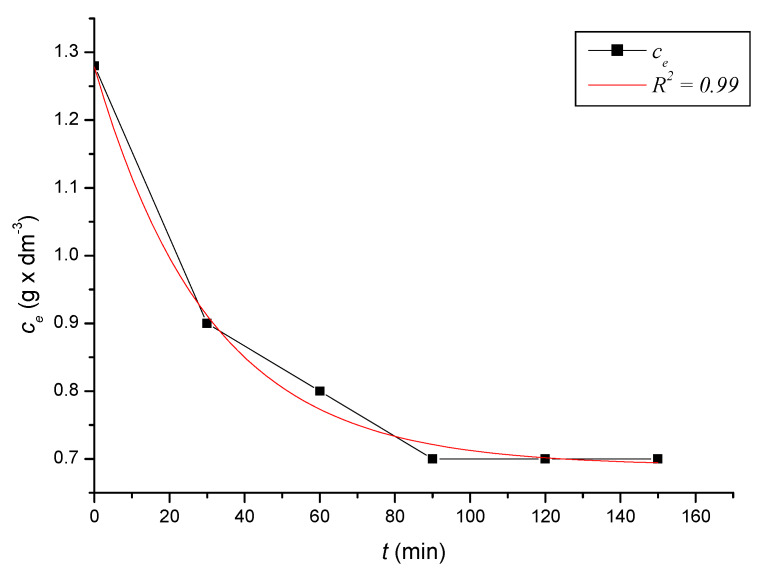
The kinetic course of reducing the concentration of Fe^3+^ cations in a solution with an initial concentration of 1.28 g × dm^−3^ by the action of clinoptilolite.

**Figure 7:**
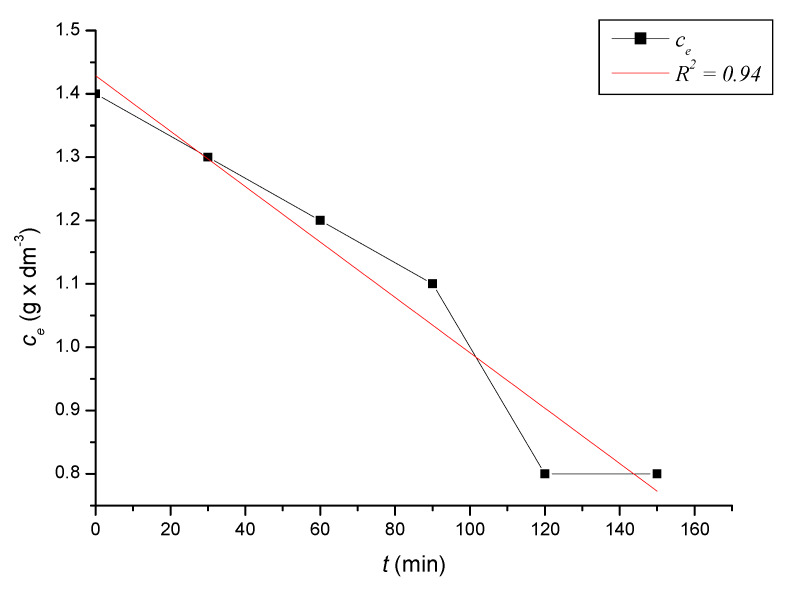
The kinetic course of reducing the concentration of Fe^3+^ cations in a solution with an initial concentration of 1.40 g × dm^−3^ by the action of clinoptilolite.

To define the time required to reach equilibrium in the system, we monitored the dependence of the adsorbed amount on the time of contact of the adsorbent with the adsorbate. At precise time intervals, we measured the content of iron cations in the solutions until the equilibrium state was reached. Based on the decreases in the content of iron cations, we calculated the absorbed amounts of iron cations on the zeolite samples. After reaching the equilibrium state of the systems, we calculated the equilibrium adsorption capacities. The kinetic course of the reduction of the content of iron cations on the sorbent in solutions with different initial concentrations was expressed graphically (Figure 2, Figure 3, Figure 4, Figure 5, Figure 6 and Figure 7) and analytically (4–9). An exponential dependence (10) applies to the dependence in Figure 2:

(4) $$c_{e}=0.068-0.407\times e^{\left(-\frac{t}{119.029}\right)}$$

An exponential dependence (5) applies to the dependence in Figure 3:

(5) $$c_{e}=0.293+0.268\times e^{\left(-\frac{t}{31.378}\right)}$$

An exponential dependence (6) applies to the dependence in Figure 4:

(6) $$c_{e}=0.399-0.421\times e^{\left(-\frac{t}{21.360}\right)}$$

An exponential dependence (7) applies to the dependence in Figure 5:

(7) $$c_{e}=0.604+0.515\times e^{\left(-\frac{t}{18.873}\right)}$$

An exponential dependence (8) applies to the dependence in Figure 6:

(8) $$c_{e}=0.690+0.589\times e^{\left(-\frac{t}{30.714}\right)}$$

An exponential dependence (9) applies to the dependence in Figure 7:

(9) $$c_{e}=-497.629+499.057\times e^{\left(-\frac{t}{114\;072.093}\right)}$$

The dependencies mentioned above declared by Equations (10) to (15) describe the dependences of the cation concentration of Fe^3+^ on time *t*, which range in the tightness diaphragm from 0.94 to 0.99.

The results were processed graphically by adsorption isotherms and mathematically evaluated using the Freundlich adsorption isotherm. The initial and measured equilibrium concentrations of the sample solutions, as well as the calculated absorbed amounts, are given in Table 1.

### 4.2. The Freundlich Sorption Isotherm and the Actual Prediction Model

**Figure 8:**
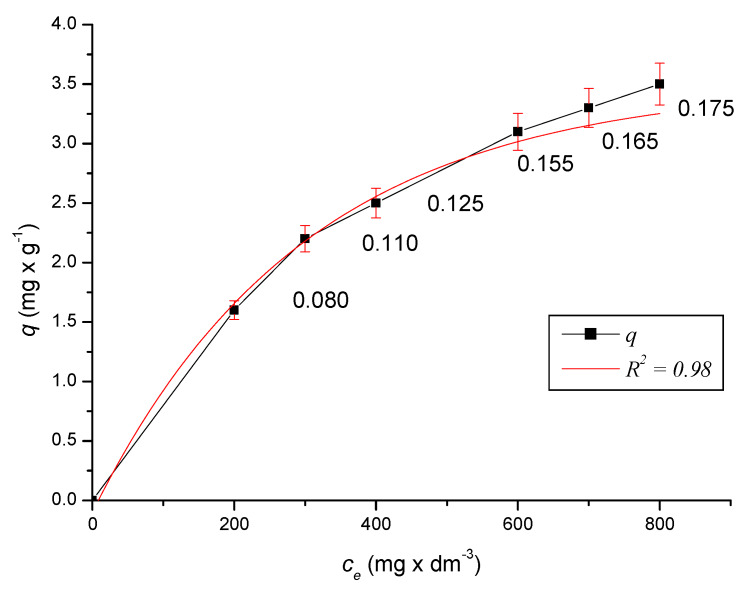
Dependence of adsorption capacity *q* on equilibrium concentration *c_e_*.

**Figure 9:**
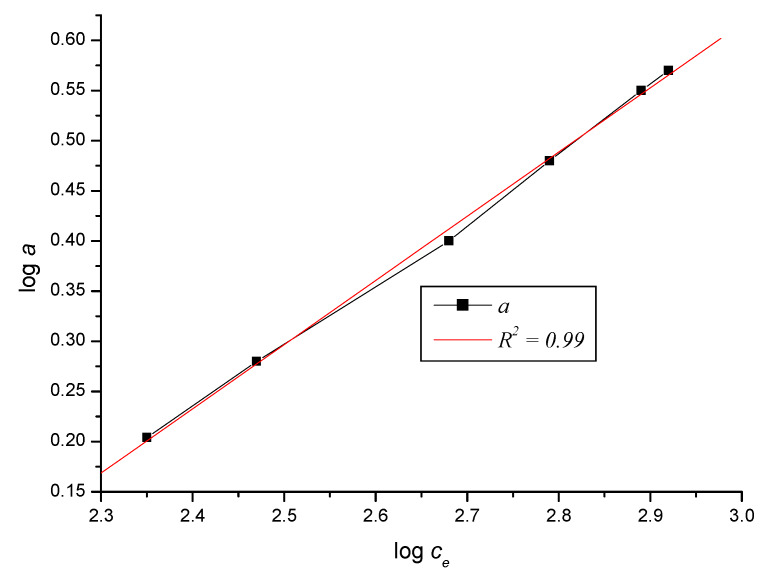
Linearization of the Freundlich isotherm for adsorption and equilibrium concentration values.

By plotting the experimentally determined values *c_e,i_*_,_ *q_i_*, the desired isotherm was determined (Figure 8). By linearization of the isotherm, it was verified which model suits the given sorption. Since the dependences of the adsorbed amounts of ferric cations on the equilibrium concentration in the logarithmic form are linear (Figure 9), it can be argued that the measured sorption isotherms conform to the Freundlich sorption isotherm *q = K_F_ × c_e_* ^1/*n*^ [29]. The coefficient *K_F_* is the Freundlich coefficient and can be considered as the adsorbed amount of cations in the sorbent at *c_e_*^1/*n*^ = 1 mg × dm^−3^, with the exponent *N* = 1/*n* being an indicator of the non-linearity of the sorption isotherm. If the Freundlich isotherm is suitable for expressing *q = f (c_e_)*, then it is possible to interpret the line by experimental values plotted in the coordinates *X = log c_e_, Y = log q* (Figure 9). The standard deviation of the three measurements is added to the graph of the dependence of the adsorption capacity on the equilibrium concentration according to Equation (10) to obtain relevant statistical data. Equation (10) presents a prediction model that is compared with the Freundlich model (Chapter 5).

For regression equation *q = q(c_e_)* with the accuracy of 0.98 (Figure 8) it is valid (10):

(10) $$q=3.700-3.791\times e^{\left(-\frac{c_{e}}{320.709}\right)}$$

For the dependence on Figure 9, the linear course declared by Equation (11) applies:

(11) $$\log q=-1.303-0.640\times \log c_{e}$$

The least-squares method was used to calculate the constants of the Freundlich isotherm. The slope *k* and the coefficient *z* were evaluated according to Equations (12)–(14):

(12) $$k=-\frac{n\times \sum_{i=1}^{n}x_{i}\times y_{i}-\sum_{i=1}^{n}y_{i}\times \sum_{i=1}^{n}x_{i}}{n\times \sum_{i=1}^{n}x_{i}^{2}-{\left(\sum_{i=1}^{n}x_{i}\right)}^{2}}$$

(13) $$z=\frac{1}{n}\times \left(\sum_{i=1}^{n}x_{i}-k\times \sum_{i=1}^{n}y_{i}\right)$$

(14) $$x=\log c_{e};\;y=\log q$$

The measured sorption data are consistent with the Freundlich sorption isotherm (15):

(15) $$q=K_{F}\times c_{e}^{\frac{1}{n}}$$

If *n* = 1/*k* and *K_F_* = 10^*z*^, then *K_F_* reaches the value of 0.034, and for coefficient *n*, the value of 1.39. Isotherm can be expressed by Equation (16):

(16) $$q=0.034\times c_{e}^{0.719}$$

The highest efficiency of *E* (56%) of sorbent-clinoptilolite was recorded based on Equation (9) for the lowest initial Fe(III) concentration. The calculated efficiencies for each initial sample concentration are given in Table 2.

## 5. Discussion

### 5.1. Comparison, Verification, and Interpretation of Obtained Data

**Figure 10:**
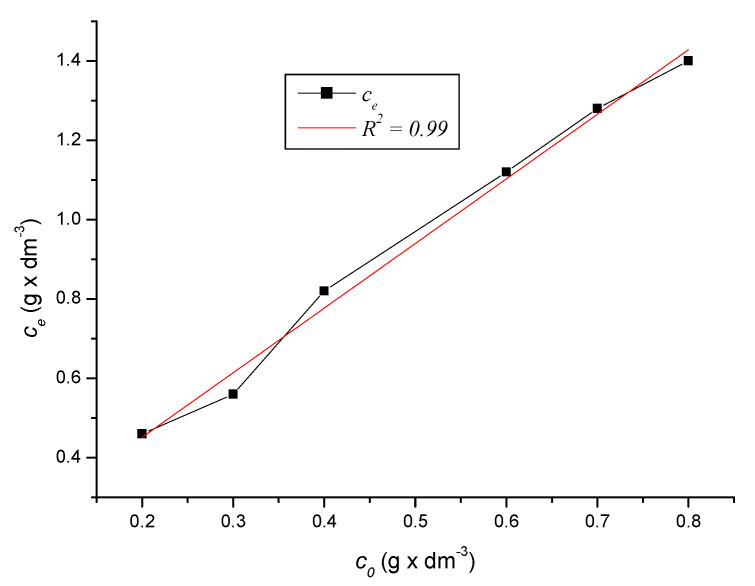
Dependence of equilibrium concentration on initial concentration in 120 min.

**Figure 11:**
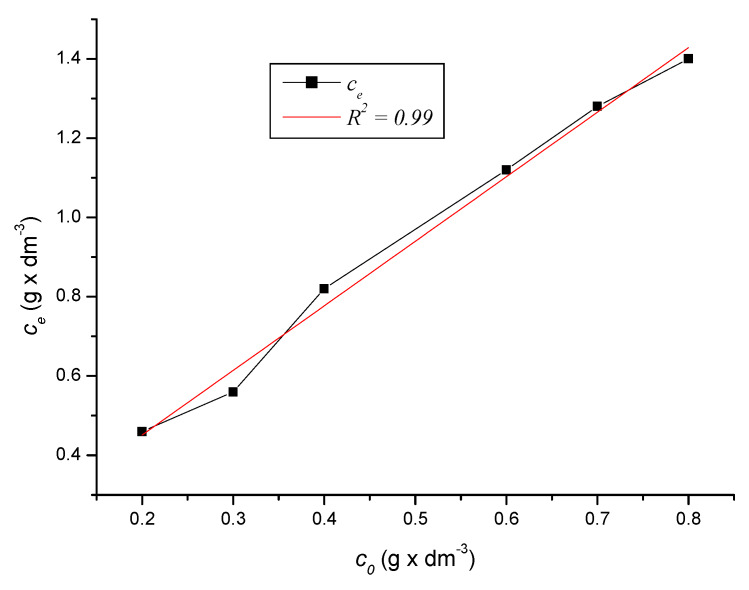
Dependence of equilibrium concentration *c_e_* on initial concentration *c*_0_ in 150 min.

Full equilibrium of the solution was reached after 90 to 120 min of measurement. The plot of this phenomenon, i.e., the dependence of the equilibrium concentration on the initial concentration of the solution, is given by the graph in Figure 10 for the period of 120 min and the graph in Figure 11 for the period of 150 min, including Equation (17).

Equation (17) applies to Figure 10 and Figure 11:

(17) $$c_{e}=-2999.134-2999.260\times e^{\left(\frac{c_{0}}{1842.236}\right)}$$

The Freundlich isotherm *q = K_F_* × *c_e_*^1/*n*^ was suitable for expressing the dependence *q = f(c_e_)*. We found this graphically, after plotting the experimentally obtained values into the coordinate system, in the coordinates *X_i_* = *c_e,i_*, *Y_i_* = *c_e,i_*/*q_i_* according to the Langmuir model of the isotherm and in the coordinates *X_i_* = log *c_e_*, *Y_i_* = log *q_i_* according to Freundlich isotherm model.

**Figure 12:**
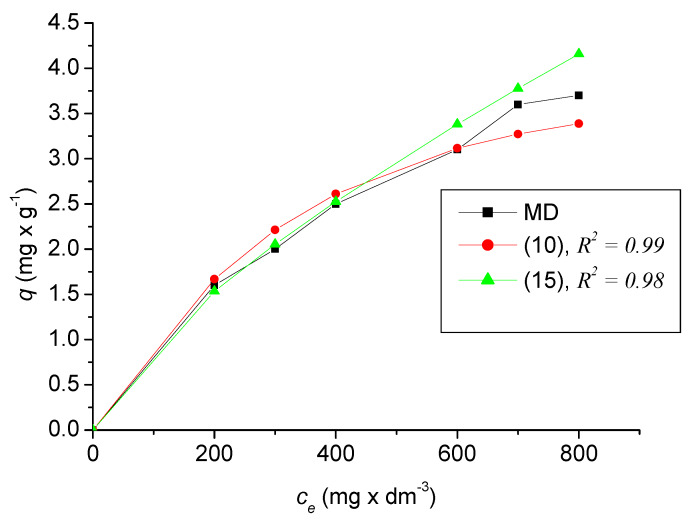
Comparison of dependences of adsorption capacity *q* on the equilibrium concentration of *c_e_* calculated by Equations (10), (15) with measured experimental data (MD).

Figure 12 compares the dependences of the adsorption capacity *q* on the equilibrium concentration of *c_e_* in the models described by Equations (10), (15) with measured experimental data (MD). As can be seen from the graphs, the data from Equation (10) are closer to the experimental data than the data from Equation (15), especially with increasing *c_e_*. This fact is also confirmed by the average values given in Table 3. The difference between the two models ∆ is also given in Table 3.

### 5.2. Calculation of the Standard Deviation

The measurements were performed three times under the same conditions. The Grubbs test excluded extreme values from the set of measured values. The lowest and highest values were tested by substituting them into Equations (18), (19), and then compared to the Grubs test table value. The standard deviations were evaluated according to Equation (20), their numerical values were determined as 0.0495, 0.0158, 0.0361, 0.0361, 0.0456, 0.0436:

(18) $$H_{\min}=\left|\frac{x_{priem}-x_{\min}}{s}\right|$$

(19) $$H_{\max}=\left|\frac{x_{priem}-x_{\max}}{s}\right|$$

(20) $$s=\sqrt{\frac{\sum_{i=1}^{n}{\left(x_{i}-x_{priem}\right)}^{2}}{n-1}}$$

The maximum adsorbed amounts *q_i_* calculated for each equilibrium concentration were compared to the adsorption capacity values calculated by the adsorption isotherm. The values of *q_i_* calculated for each measured equilibrium *c_ri_* concentration, together with the values (*q_i_* − *q_v_*)^2^ required for the calculation of the standard deviation, are given in Table 4.

The magnitude of the error is expressed by the standard deviation according to Equation (21):

(21) $$s=\sqrt{\frac{{\left(q_{i}-q_{v}\right)}^{2}}{n-2}}$$

where *q_i_* represents the measured adsorption capacity values, and *q_v_* the adsorption capacity values calculated according to the respective adsorption isotherm. The standard deviation was determined according to Equation (21) *s* = 0.3242 mg × dm^−3^. There were only minimal differences between the maximum adsorbed amounts *q_i_* and between and *q_v_* values calculated according to the adsorption isotherm (Table 4). The kinetic curves show that the optimal time to reach equilibrium with natural zeolite-clinoptilolite is at least 90 min. Sorption efficiency was determined to be in the range of 43% to 56%. In two model samples, with an initial concentration of 0.46 g × dm^−3^ (Figure 2) and 1.40 g × dm^−3^, there was a decrease after 120 min to a concentration of 0.20 g × dm^−3^ and to 0.8 g × dm^−3^. The other model samples reached equilibrium after 90 min when we recorded a decrease from concentrations of 0.56 g × dm^−3^ to 0.30 g × dm^−3^ (Figure 3), 0.82 g × dm^−3^ to 0.40 g × dm^−3^ (Figure 4), 1.12 g × dm^−3^ to 0.60 g × dm^−3^ (Figure 5), 1.28 g × dm^−3^ to 0.70 g × dm^−3^ (Figure 6), to 1.40 g × dm^−3^ to 0.80 g × dm^−3^ (Figure 7). Due to these facts and since clinoptilolite is a cheap natural, non-toxic material, its more extensive use for the treatment of drinking water and wastewater is promising.

The reduction of iron content by biosorption on iron-reducing bacteria has been discussed by the authors of the publication [30]. At an initial concentration of 2 g × L^−1^, the amount absorbed approached 536.1 ± 26.6 mg Fe(III) (g × EPS)^−1^. In the article [16], the authors investigated the effectiveness of surface-treated zeolites with MnO called Klinopur—Mn and Klinomangan. The average iron ion concentration of 3.92 mg × L^−1^ of the model samples was reduced below the limit value of 0.2 mg × L^−1^. We determined sorption capacities on natural clinoptilolite, the value of which increased in proportion to the concentration of iron in the solution. We compared the measured values of sorption capacities with the calculated values after substituting into the obtained sorption isotherm, in which event we can state that the differences were minimal. The determined sorption capacities ranged from 1.6 mg × g^−1^ to 3.7 mg × g^−1^.

**Figure 13:**
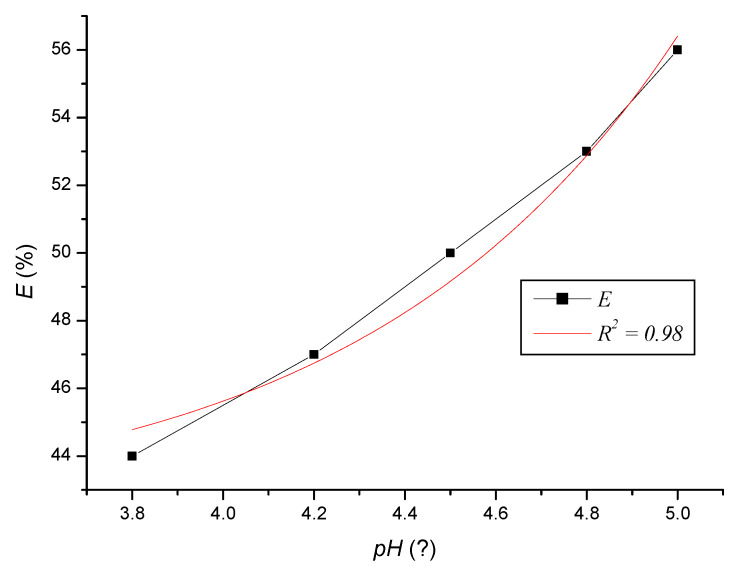
Dependence of the evaluated sorbent-clinoptilolite activity *E* on the pH value of wastewater.

We supplemented the presented experiments with the effect of Zeolites are regenerated on the efficiency of the sorbent (Table 5). The calculated values of clinoptilolite efficiency for the pH range 3.8–5.0 are expressed by Equation (22) and are shown in Figure 13.

(22) $$E=42.238+0.011\times e^{\left(-\frac{pH}{0.698}\right)}$$

In the range of pH 3.8 and 5.0, the highest efficiency of the sorbent was, i.e., also the sorption capacity at pH 5, but the differences between the individual activities were small. At higher pH values, the negative charge of the sorbent increases, which aids in the adsorption of cations present in the solution. At low pH values, the sorption of metal cations is relatively low due to competition for sorption sites with a proton (H^+^). At present, various materials based on natural zeolite are being tested in view of the increasing ecological needs applied in practice. Clinoptilolite can also be surface functionalized by various chemical treatments, thus increasing its sorption capacity. Contact filtration through a suitable material is economically acceptable and undemanding iron removal technology [27]. The pH value = 5 was determined for the presented experiments on the basis of Table 5, because it represents a slightly acidic environment in which Fe^3+^ ions bind well.

Although the ion exchange method has many advantages, the used zeolites must be disposed of or regenerated. The reference [31] shows that zeolites can be almost completely regenerated with NaCl regeneration solution at pH > 12. At present, the biological method [32,33,34], which is environmentally friendly and less energy-intensive, is used to regenerate zeolite.

## 6. Conclusions

Sorption is a promising and cost-effective technology for removing ions from aqueous solutions. Compared to conventional methods, it has several advantages, namely low operating costs, high efficiency in removing lower concentrations, ion removal in a relatively short time. Current research on sorption of heavy metals focuses mainly on the use of natural, non-toxic materials. Sorbents for environmental protection use adsorption more efficiently than absorption. In the case of adsorption, the liquid is more firmly bound to the sorbent surface, and possible resorption would be difficult.

The novelty and contribution of the presented experiments to the practice lie mainly in the fact that natural, non-toxic zeolite-clinoptilolite material was used to reduce the concentration of iron cations, which could replace synthetic cost-effective sorption materials. The Freundlich isotherm can describe the development of the sorption process of iron cations (III). It was evaluated and verified that the measured sorption isotherms comply with the Freundlich sorption isotherm. Isotherm parameters were calculated using experimental results of adsorbed ferric cations per gram of sorbent versus their equilibrium concentrations in solution. The searched adsorption isotherm has analytical form. The isotherm was verified experimentally by regression equation *q* = *q* (*c_e_*) with the accuracy of 0.98. Based on our goal, we obtained a new prediction model. We compared this model with the measured data (MD) and with the classical Freundlich model.

From kinetic dependencies, it was shown that the optimal time for equilibration in the adsorption system is 90 min. In most cases, the equilibrium concentration was achieved after 90 min, and in one case, after 120 min. Subsequently, the dependence of the achieved equilibrium concentration on the initial concentration of the solution was expressed, namely graphically and analytically. Increasing the effectiveness of natural zeolite by surface treatment will be the subject of further research (sorption efficiency was determined in the range 42.57%–56.00%).

Based on the presented research, adsorption isotherms tested in laboratory conditions can be recommended for subsequent preliminary investigation of possible technological use of natural zeolite in the process of drinking and wastewater treatment. Experimental research into the sorption removal of heavy metals in drinking and wastewater treatment will continue, also with a focus on research into the modified natural zeolite.

## Figures and Tables

**Table 1:** Equilibrium concentrations of Fe^3+^ in solutions and adsorbed amounts relative to different initial concentrations.

*c*_0_ (g × dm^−3^)	*c_e_* (g × dm^−3^)	*q* (mg × g^−1^)
0.46	0.20	1.60
0.56	0.30	1.90
0.82	0.40	2.63
1.12	0.60	3.01
1.28	0.70	3.60
1.40	0.80	3.70

**Table 2:** Equilibrium concentrations of Fe^3+^ in solutions and adsorbed amounts relative to different initial concentrations.

*c*_0_ (g × dm^−3^)	*c_e_* (g × dm^−3^)	*E* (%)
0.46	0.20	56.00
0.56	0.30	43.00
0.82	0.40	51.21
1.12	0.60	46.40
1.28	0.70	45.31
1.40	0.80	42.60

**Table 3:** Comparison of models with measured data (MD) and their mutual differences.

*c_e_* (mg × dm^−3^)	*q* MD (mg × g^−1^)	*q* (10) (mg × g^−1^)	*q* (15) (mg × g^−1^)	∆
0	0	0	0	0
200	1.6	1.668	1.534	−0.134
300	2	2.212	2.054	−0.159
400	2.5	2.611	2.526	−0.085
600	3.1	3.116	3.380	0.264
700	3.6	3.273	3.777	0.504
800	3.7	3.387	4.157	0.770
	Ø 2.371	Ø 2.324	Ø 2.490	Ø 0.166

**Table 4:** The adsorbed amount and calculated adsorption values.

*c_ei_* (mg × dm^−3^)	*q_i_* (mg × g^−1^)	*q_v_* (mg × g^−1^)	(*q_i_* − *q_v_*)^2^
200	1.60	1.53	0.0049
300	1.90	2.05	0.0225
400	2.63	2.52	0.0121
600	3.01	3.38	0.1369
700	3.60	3.78	0.0324
800	3.70	4.16	0.2116

**Table 5:** Efficiency of clinoptilolite depending on the pH value of the wastewater.

pH (−)	Efficiency (%)
3.8	44
4.2	47
4.5	50
4.8	53
5.0	56
